# Better care provided to patients with tuberculosis at county designated TB hospitals (CTD) compared to non-CTDs in rural China

**DOI:** 10.1186/s12879-016-2108-8

**Published:** 2017-01-13

**Authors:** Li Yuan, Hui Zhang, Changming Zhou, Weili Jiang, Qi Zhao, Xu Biao

**Affiliations:** 1 Department of Epidemiology, School of Public Health, Fudan University, 138 Yi Xue Yuan Rd, Shanghai, 200032 China; 2 Key Laboratory of Public Health Safety (Ministry of Education), 138 Yi Xue Yuan Rd, Shanghai, 200032 China; 3 National Center for TB Control and Prevention, National center for TB control and prevention, 155 Changbai Road, Beijing, 102206 China; 4 Centre for Global Health, Department of Public Health Science (IHCAR), Karolinska Institutet, S-171 77 Stockholm, Sweden

**Keywords:** Tuberculosis, Re-treated, County TB dispensary, General health facility, China

## Abstract

**Background:**

The primary unit of tuberculosis (TB) medical care in China is the county TB dispensary or county designated hospital (CTD), where patients can receive free diagnosis and treatment. However, a substantial number of patients seek their anti-TB treatment from general health facilities (Non-CTDs). This study aimed to investigate the first anti-TB treatment experience and choice of health facilities of retreated TB patients and their determinants.

**Methods:**

A cross-sectional study was conducted in Jiangsu, Shandong and Sichuan provinces. All registered re-treated TB patients were investigated using a structured questionnaire covering information on demographics, socio-economic characteristics, and previous anti-TB treatment experiences.

**Results:**

Totally, 75.3% of 544 patients visited CTD directly for initial treatment. Patients who were female (OR:1.71, 95% CI: 1.01-2.87), over 40 years of age (OR:2.80, 95% CI: 1.24-6.33), from Jiangsu (OR:3.07, 95% CI: 1.57-6.01) and Sichuan (OR:4.47, 95% CI: 2.29-8.73) and those diagnosed before 2005 (OR:6.87, 95% CI: 4.24-11.13) had a significant higher risk receiving their initial treatment at a non-CTD. Patients were more likely to have standardized diagnosis and treatment regimens in CTD (89.8%) than in non-CTDs (65.9%). Patients treated in non-CTDs versus in CTD had a lower possibility to complete their treatment course during first TB episode (*χ* ^2^ = 3.926, *P* = 0.048), but there was no significant difference in the cure rate between different facilities (CTD 60.8%, Non-CTDs 59.1%). Patients in non-CTDs incurred higher costs (1,360 CNY) than those treated in CTD (920CNY).

**Conclusion:**

CTD play a key role in the National Tuberculosis Control Program. Patients should be guided to seek health care in county designated hospital, where they are more likely to receive appropriate examinations, treatment regimens and rigorous supervision, and to bear a lighter economic burden.

## Background

Tuberculosis (TB) remains a major global health problem, responsible for ill health among millions of people each year [1]. TB is one of the world’s deadliest communicable diseases. There were 9.0 million new TB cases in 2014 and 1.5 million TB deaths, including 320,000 deaths among HIV-positive people [1]. With the third heaviest burden of TB worldwide over the past two decades, China holds 12% of global TB patients [1]. China’s National TB control Program started to implement the international recommended directly observed treatment, short-course (DOTS) strategy in 1991, and expanded the DOTS programme to the entire country by 2005 [2]. Over the past 20 years, the prevalence and mortality rates of TB has been reduced by half. The estimated smear-positive prevalence decreased from 170/100,000 in 1990 to 59/100,000 in 2010 and the mortality rate declined rapidly at an average rate of 8.6% per year during the period between 1990 and 2010 [3, 4].

Nevertheless, China still faces daunting challenges and difficulties in TB control, and a rapid multi-drug-resistant (MDR) TB increase is one of them. There are approximately 120,000 new MDR-TB cases annually in China, accounting for 24% of the global MDR-TB burden [5]. Several studies have shown that pervious treatment is the strongest determinant of MDR-TB [6–8]. The acquired drug-resistant TB mainly result from incompliance to treatment, uncompleted treatment, irregular treatment and unqualified health services. A study in Moldova found that 7.2-9.2% patient turned from non-MDR-TB to MDR-TB during their treatment course, likely due to unregulated treatment and nosocomial transmission [9].

The administration structure in rural China is three tiers from village to town to county. There were about 2800 counties in total according toNew Statistical Year books published by National Bureau of Statistics of China in 2016 [10]. In general, each county has about 10–15 townships and each township administers 10–20 villages. The health service in rural is also a three-tier system, with 1–2 county-level hospitals, one township hospital or health center in township level and village health station in each village. The basic unit of TB health care in China is the specialized County TB dispensary or County designated TB clinics (CTD), which has been set in more than 2500 Chinese counties. The CTDs take the responsibilities of TB diagnosis, treatment and patient management guided by the National TB control program. TB diagnosis is microscopy-based following the guideline of the WHO and IUTLD. The recommended treatment regimen for new TB case and retreated TB cases were 2HRZS/4HR and 2HRZSE/6HRE [11].

However, it is common in rural areas that patients initiated their health care seeking for cough and other TB symptoms in general health facilities instead of CTD, such as village health stations, township and county hospitals. Most of these health facilities (non-CTDs) don’t have the capacity for TB treatment. The non-CTD’s role in China’s TB control program is to refer suspected TB patients to CTDs for standardized diagnosis, treatment and management. It is unclear whether TB patients are treated and managed equally well in the two types of facilities. Our study investigated the first anti-TB treatment experience and choice of health facilities of retreated TB patients and their determinants in three provinces.

## Study population and methods

### Study Setting

Three provinces were purposively selected for the consideration of geographic and economic variation in 2012 and 2013. Shandong and Jiangsu are plain provinces in eastern China while Sichuan is a province in western China with vast mountainous areas. In 2013, the populations of Jiangsu, Shandong, and Sichuan Provinces were 79.39, 97.33, and 81.07 million respectively. The average annual income for rural residents was 1,739 US Dollars (USD) per capita in Jiangsu, 1,343 USD in Shandong and 987 USD in Sichuan, while the national average was 1,123 USD in 2011 [12]. Eleven counties, 4 from Jiangsu province, 3 from Shandong, and 4 from Sichuan province were selected covering a population of 5, 4 and 4 million respectively in the three provinces.

### Data collection

All registered 544 active pulmonary TB patients with a history of at least one anti-TB treatment were investigated after written informed consent was obtained. Among them, 200 were from Jiangsu, 149 from Shandong and 195 from Sichuan.

Physicians, who had undergone a 1-day training course for the study, interviewed the subjects at the time of TB diagnosis.

A structured questionnaire was applied covering informationon general demographic and social-economic characteristics, disease history, previous anti-TB treatment experiences, TB related health expenditures and disease profile. Expenditures on TB related health care during the whole treatment course were self-reported by patients. Direct medical costs included expenditures onexaminationsand testing, medication and hospitalization, indirect costs were expenditures on transportation, accommodation, food and other related costs for each visit.

### Data analysis

Data were double entered with EpiData 3.1 (Denmark) and analyzed using SPSS for Windows version 16.0 (SPSS, Chicago, IL, USA). Descriptive statistics were applied including medians, means, proportions and frequencies. Either Chi-square test or Mann–Whitney U test were used in univariate analyses to identify factors associated with the outcomes of interest. A logistic regression model was used to examine the associations of interest and adjust odds ratios (aOR) and 95% confidence intervals (95% CIs) were calculated.

## Results

### General characteristics of subjects

A total of 544 previously treated TB patients were enrolled in the study, including 402 (74.2%) male and 140 (25.8%) female patients, 17 to 98 years of age (mean: 55.29, standard deviation: 15.33 years). Of them, 383 (70.5%) were farmers and 339 (62.7%) had no more than 6 years of education; 531 (98.2%) had medical insurance, mainly covered by New Rural Cooperative Medical Insurance (90.2%).

### Diagnosis and treatment experience

**Fig. 1:**
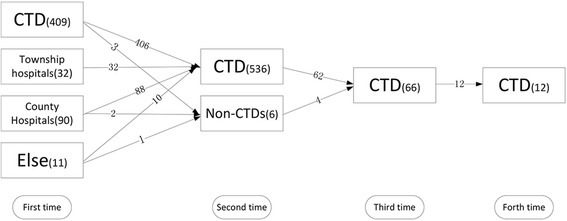
Pathways of health facilities during previous anti-TB episodes

A majority of the TB patient had one previous anti-TB treatment episode, 10.3% had twice and 2.2% had three times before investigation. Among the patients, 380 (70.1%) received their first TB diagnosis after 2005 when the DOTS strategy had been adopted widely in the country. Three quarters (75.3%, 409/544) of the patients were treated in the CTD for their first treatment episode, and others were treated in either township hospitals (5.9%, 32/544) or county hospitals (16.5%, 90/544). For the patients who had more than one treatment episode, nearly all the patients who were treated in township (100%, 32/32) or county hospitals (98.5%, 88/90) first time had a referral to CTD and totally 98.5% (536/544) of the patients chosen CTD for their second episode (Fig. 1).

Logistic regression analysis was applied to determine the associations of the selection of treatment facilities with patients’ demographics, socio-economic status, and times of treatment episodes. It was found that gender, age, region, first diagnosis year and average annual income were significantly associated with the selection of treatment facilities. Compared to other patients, increased odds for visiting a non-CTD for their first course of anti-TB treatment were observed in females (OR: 1.71, 95% CI:1.01-2.87), those more than 40 years of age (OR: 2.80, 95% CI:1.24-6.33), those from Jiangsu (vs. Shandong, OR: 3.07, 95% CI:1.57-6.01) and Sichuan (vs. Shandong, OR: 4.47, 95% CI: 2.29-8.73), and those diagnosed before 2005 (OR: 6.87, 95% CI: 4.24-11.13).No statistically significant associations were found with the times of treatment episodes, marital status, education level, occupation and medical insurance status of patients (Table 1).

**Table 1:** Logistic regression analyses for determinants affecting patients’ choices of health facilities (Non-CTD vs. CTD)

Categories	CTDs	Non-CTDs	cOR (95% CI)	aOR (95% CI)
	No (%)	No (%)		
Gender (female/male)				
Male	308 (76.62)	94 (23.38)	1.357 (0.882-2.088)	1.705 (1.014-2.866)
Female	99 (70.71)	41 (29.29)		
Marriage (unmarried/married)				
Married	363 (74.08)	127 (25.92)	2.566 (1.068-6.164)	1.342 (0.477-3.774)
Unmarried	44 (88.00)	6 (12.00)		
No. of previous TB episodes				
1	354 (74.37)	122 (25.63)		
2 (2 vs.1)	47 (83.93)	9 (16.07)	0.556 (0.265-1.167)	0.451 (0.197-1.034)
3-4 (3–4 vs.1)	8 (66.67)	4 (33.33)	1.451 (0.429-4.903)	1.868 (0.460-7.574)
First diagnosis year* (before/after)				
Before 2005	83 (51.23)	79 (48.77)	5.507 (3.623-8.371)	6.869 (4.241-11.127)
After 2005	324 (85.26)	56 (14.74)		
Education degree (≥6/<6)				
< 6 years	251 (74.04)	88 (25.96)	0.865 (0.576-1.299)	0.825 (0.490-1.392)
≥ 6 years	155 (76.73)	47 (23.27)		
Insurance (insured/non)				
Non-insured	7 (70.00)	3 (30.00)	0.766 (0.195-3.005)	0.682 (0.148-3.143)
Insured	402 (75.28)	132 (24.72)		
Income quartile#				
Group 1	111 (77.62)	32 (22.38)	1.077 (0.626-1,852)	0.922 (0.491-1.730)
Group 2 (2 vs.1)	116 (76.32)	36 (23.68)		
Group 3 (3 vs.1)	94 (73.44)	34 (26.56)	1.255 (0.720-2.186)	1.105 (0.569-2.147)
Group 4 (4 vs.1)	73 (69.52)	32 (30.48)	1.521 (0.858-2.694)	1.893 (0.923-3.882)
Occupation (farmer/non)				
Non-farmer	119 (74.38)	41 (25.63)	0.944 (0.618-1.443)	1.222 (0.698-2.138)
Farmer	289 (75.46)	94 (24.54)		
Age (≥40/<40)				
< 40 years old	73 (86.90)	11 (13.10)	2.509 (1.288-4.887)	2.800 (1.239-6.328)
≥ 40 years old	328 (72.57)	124 (27.43)		
Provinces				
Shandong	130 (87.25)	19 (12.75)		
Jiangsu (JS vs.SD)	151 (75.50)	49 (24.50)	2.220 (1.244-3.963)	3.070 (1.568-6.010)
Sichuan (SC vs. SD)	128 (65.64)	67 (34.36)	3.581 (2.036-6.300)	4.467 (2.285-8.734)

### Laboratory examinations and chemotherapy regimens during first anti-TB treatment episode

Chest X ray examination (92.65%) and sputum smear test (81.80%) were mostly commonly used for TB diagnosis. Among the 113 patients treated in non-CTDs, 48.89% received sputum smear tests, and the proportion was significantly lower than the patients treated in CTD (379 and 92.67%, *P* < 0.001). A trend was also observed for the proportion of patients receiving sputum culture or drug sensitive test (*P* < 0.001). On the contrary, patients treated in non-CTDs had a higher possibility to receive chest fluoroscopy (*P* < 0.001), blood routine tests (*P* = 0.033) and Computerized Tomography (CT) test (*P* < 0.001) (Table 2).

**Table 2:** Laboratory examinations during first anti-TB episode

Examinations	Total number	CTD			Non-CTD		*P*
		N	%		N	%	
Sputum smear test	445	379	92.67		66	48.89	<0.001
Sputum culture/Drug sensitive test	73	67	16.38		6	4.44	<0.001
Chest fluoroscopy	123	67	16.38		56	41.48	<0.001
Chest X ray examination	504	379	92.67		125	92.59	0.978
Blood routine test	255	181	44.25		74	54.81	0.033
CT	107	59	14.43		48	35.56	<0.001

Totally, 87 patients (65.9%), who were treated in non-CTDs during their first anti-TB treatment episode, received standardized regimen. The proportion was significantly lower than those treated in CTD (89.8%, *P* < 0.001) (Table 3). About 16.1% were treated using loose drugs or other combinations. It was found that some patients may only take isoniazid and rifampicin while treated in non-CTDs, or may had a treatment course shorter or longer than the standardized duration, or received 2nd line anti-TB drug without drug susceptibility testing. It was found that 36 of the 423 patients (8.5%) had received 2nd-line anti-TB drugs during their initial treatment. The most common drug was fluoroquinolone, which was used by 33 patients.

**Table 3:** Treatment regimen and outcome of initial treatment

Characteristics		Non-CTD	CTD	*Total*	*P*-value^*^
		No. (%)	No. (%)	No. (%)	
Regimen	2HRZS/4HR^+^	87 (65.9)	362 (89.8)	449 (83.9)	<0.001
	Others	45 (34.1)	41 (10.2)	86 (16.1)	
Outcome	Cure	68 (59.1)	240 (61.2)	308 (60.7)	<0.001
	Treatment completed	0 (0.0)	37 (9.4)	37 (7.3)	
	Treatment failed	21 (18.3)	57 (14.5)	78 (15.4)	
	Treatment interrupted	26 (22.6)	58 (14.8)	84 (16.6)	
	Medium length of treatment (p25, p75) (M)	6 (5,7)	6 (6,6)	6 (5,7)	0.279^**^

+ Recommended treatment regimen for new TB case

* Chi-square test

** Mann–Whitney U test

### Treatment adherence of patients during first anti-TB treatment episode

Most patients (83.4%, 423/507) completed their first treatment course and 60.4% (304/503) were cured after the treatment (Table 3). The accomplishment proportions of patients treated in CTD and Non-CTDs were 85.2% and 77.4%, respectively; Patients treated in non-CTDs had a lower possibility to complete their treatment course during first TB episode (*P* = 0.048), but the cure rates showed no significant difference between CTD (60.8%) and non-CTDs (59.1%). Among the 84 patients who had an interruption for their treatment course, 55.6% perceived themselves to be healthy and felt no need to complete the course; 21.0% gave up due to serious side effects of anti-TB drugs. Other reasons for treatment interruption included heavy economic burden (16.0%) and moving to other regions (3.7%).

### Economic burden for patients during first anti-TB treatment episode

Our study showed that the average financial burden on patients was 2382 CNY (median 1,012 CNY, [IQR 500–2968 CNY]). Half of the patients incurred costs of more than 17% of their individual annual income and over 7% of their annual family income. The median financial burden on patients treated in CTD and non-CTDs was 920 CNY [450–2,800 CNY] and 1,360 CNY [720–3,000 CNY], respectively (Table 4).

**Table 4:** Economic burden for patients during first anti-TB episode

Cost (CNY)	Response No.	CTD (Median, IQR)	Non-CTD (Median, IQR)	P*
Total	358	1055 [505,3700]	2030 [1028,3016]	0.001
Medical costs	504	1000 [400,3000]	2000 [600,3000]	0.001
Non-medical costs	359	63 [20,420]	30 [1,100]	0.011
Transportation	498	16 [10,30]	16 [1,30]	0.803
Accommodation and food	424	10 [0,20]	0 [0,20]	0.032
Other costs	364	10 [0,200]	0 [0,20]	<0.001
Covered by insurance	393	0 [0,800]	0 [0,800]	0.149
Patient financial burden	352	920 [450,2800]	1360 [720,3000]	0.003

*Mann–Whitney U test

## Discussion

The basic principles of care for TB patients are the same worldwide: a diagnosis should be established promptly and accurately; standardized treatment regimens of proven efficacy should be used with appropriate treatment support and supervision; and the response to treatment should be monitored [13].

In China, the primary TB medical care unit is the CTD, where patients receive free diagnosis and treatment. But our study showed that there were still some patients who visited non-CTDs for their anti-TB treatment. In our study, only 75.3% of the subjects chose CTDs for anti-TB treatment during their first episode, and the rest of patients received anti-TB services from non-designated health care facilities. Patients who were female, elderly, had higher income and were diagnosed before 2005 were more likely to visit non-CTDs [14]. Previous studies also demonstrated that female and elderly patients tended to visit hospitals nearby or delay seeking for help, and individuals with higher income preferred high-ranking comprehensive hospitals [15–17]. The phenomenon that patients didn’t seek service from CTD before 2005 may have resulted from the low popularization rate of DOTS strategy.

For the quality of TB care, one surprising finding was that the patients treated in non-CTDs had a lower possibility to receive proper laboratory examinations for diagnosis. Sputum smear microscopy, the primary method for detecting TB, is required for each suspect. However, only 48.89% of the patients treated in non-CTDs received such examination. Lack of bacteriological confirmation in non-CTDs was also reflected by a low proportion of patients who received sputum culture tests or drug sensitive tests. Because the bacteriological confirmation of TB is essential for TB diagnosis, patients treated in non-CTDs would have a worse scenario than those treated in CTDs [18].

Our study also revealed that non-CTDs were less likely to provide standardized therapeutic strategy and treatment supervision compared with CTD. The TB component of the infectious and endemic disease control (IEDC-TB) project in the People’ Republic of China indicated that new smear-positive cases should be treated with 2HRZS/4HR regimen [11, 19]. Patients treated in non-CTDs had a lower possibility to obtain the standardized chemotherapy regimens than those treated in CTD and a previous study by Chinese National Center for TB Control and Prevention showed similar results that only a small proportion of TB cases in hospitals (non- CTDs) were treated with appropriate NTP/WHO treatment regimens [20]. As improper therapeutic strategy could result in an extra burden not only to the patients but also to the community by increasing chances of drug resistance development, non-CTDs were proved to be less efficient in TB control in this respect. Meanwhile, only 77.4% cases treated in general hospitals completed their first treatment course; more than half of the patients who quitted the course felt good about themselves and stopped the treatment without doctors’ permission, indicating that lacking of supervision could led to a lower rate of treatment completion in general medical institutions.

The medium financial burden and medical cost in non-CTDs tended to be higher compared with those in CTD, although the differences were not statistically significant. The exemption policy of TB treatment is only provided to the patients who have smear positive results from CTD. In addition, there is an increased hospital dependence on fee-for-service revenue, which promotes doctors in non-CTDs to provide additional clinical services and prescribe more drugs in order to maximize their profits [21]. TB has been regarded as a poverty-related disease, as it disproportionately affects the most economically disadvantaged strata of society [22]. Previous studies have documented that high financial burden is an important influencing factor for patient health care seeking behavior [23, 24] and may deter patients from seeking treatment [25].

This study had several limitations. First, only 11 counties from three provinceswere selected in the study, and therefore, a caution should be taken when extrapolating the results of this study. Second, we only studied the patients who were registered in CTD, and patients who did not register might behave differently in seeking health care. Third, information on income, expenditure, and time was based on self-reporting, and there might be recall bias, although trained interviewers tried to obtain information from different sources to reduce the bias.

## Conclusions

In conclusion, during the past twenty years, CTD played an important role in the national TB control programme, and provided better TB care services as compared to non-CTDs. With the progress of China’s TB control program and improvement of health service, TB patients should be guided to the county designated hospitals or clinics, where they are more likely to receive specialized examinations, appropriate treatment and rigorous supervision, and to bear a lighter economic burden. Findings from this study suggest that potential TB patients in rural area should be targeted for tailored health education on TB related health policy, especially the important role of TB designated hospital.

## Abbreviations

aOR: Adjust odds ratios
CIs: Confidence intervals
CTD: County TB dispensary or county designated hospital
DOTS: Directly observed treatment, short-course strategy
MDR: Multidrug-resistant
Non-CTDs: General health facilities
TB: Tuberculosis
USD: US Dollars
